# Is There an Association between Long-Term Sick Leave and Disability Pension and Unemployment beyond the Effect of Health Status? – A Cohort Study

**DOI:** 10.1371/journal.pone.0035614

**Published:** 2012-04-25

**Authors:** Hanna Hultin, Christina Lindholm, Jette Möller

**Affiliations:** 1 Karolinska Institutet, Department of Public Health Sciences, Division of Public Health Epidemiology, Stockholm, Sweden; 2 Karolinska Institutet, Department of Clinical Neuroscience, Division of Insurance Medicine, Stockholm, Sweden; The University of Hong Kong, Hong Kong

## Abstract

**Background:**

Studies have shown that long-term sick leave is a strong predictor of disability pension. However, few have aimed to disentangle the effect of sick leave and of health status.

The objective of this study was to investigate whether there is an association between long-term sick leave and disability pension and unemployment, when taking health status into account.

**Methods/Principal Findings:**

The study was based on the Stockholm Public Health Cohort, restricted to 13,027 employed individuals (45.9% men) aged 18–59 in 2002 and followed until 2007.

Hazard ratios (HR) with 95% Confidence Interval (CI) were estimated by Cox regression models adjusting for socio-demographic factors and five measures of health status.

Having been on long-term sick leave increased the risk of disability pension (HR 4.01; 95% CI 3.19–5.05) and long-term unemployment (HR 1.45; 95% CI 1.05–2.00), after adjustment for health status. The analyses of long-term sick leave due to specific illness showed that the increased risk for long-term unemployment was confined to the group on sick leave due to musculoskeletal (HR 1.70 95% CI 1.00–2.89) and mental illness (HR 1.80 95% CI 1.13–2.88) and further that there was an increased risk for short-term unemployment in the group on sick leave due to mental illness (HR1.57 95%CI 1.09–2.26).

**Conclusions/Significance:**

Long-term sick leave increases the risks of both disability pension and unemployment even when taking health status into account. The results support the hypothesis that long-term sick leave may start a process of marginalization from the labor market.

## Introduction

Despite the differences in national social security systems regarding the allowed length of sick leave, requirements for disability pension and benefit levels, studies from a variety of countries and settings have shown that long-term sick leave is a strong predictor of disability pension [1], [2], [3], [4], [5], [6], [7], [8], [9], [10], [11], [12], [13], [14], [15]. The requirement for disability pension is usually work incapacity due to a chronic disease. According to the Swedish Social Security System an individual can obtain disability pension compensation if having a work capacity which is permanently reduced by at least 25% due to illness or another reduction of one's physical or mental capacity. Hence, it seems to be unquestionable that disability pension in most cases is preceded by long-term sick leave, but the association between sick leave and disability pension is complex. The risk of disability pension varies with disease and severity of disease, and also with age, sex and socioeconomic factors [6], [16], [17], [18], [19], [20].

Moreover, specific diseases and self-rated health have been studied as determinants of disability pension, however without information about sick leave [5], [21], [22], [23]. Bültmann et al [21] suggested further investigation of the impact of sick leave on the results.

Attempts to adjust for underlying disease have been done in some studies of determinants of disability pension and early retirement with a focus on job characteristics and social class [16], [24], [25]. These showed an increased risk of disability pension with higher work load and low job control, after adjustments for health [16], [24], [25].

Sick leave may theoretically have both negative and positive consequences for the individual in terms of health and work capacity, but also in terms of aspects such as career possibilities, working conditions, social life, and life style [26]. Previous studies suggest that being on long-term sick leave may have inherent consequences on an individual's psychological well-being, work situation and social activities [27], [28]. The study by Floderus et. al. [27] indicate that long-term sick leave may lead to decreased social involvement at work and in associations and clubs as well as with the family. Furthermore long-term sick leave was also reported to a negatively affected self-image and decreased psychological well-being. In a study by Bryngelson [29] it was reported that long-term sick leave increased the risk of having economic difficulties. Social and economic marginalization may affect the vulnerability and future health of individuals thereby increasing the risk of disability pension and unemployment. However, a systematic literature review concluded that the basis was too poor for scientific evidence regarding consequences of sick leave [26]. Furthermore, none of the studies reviewed have disentangled consequences of long-term sick leave from the consequences of the underlying diseases.

Previous studies have also indicated that long-term sick leave increase the risk of unemployment [8], [30] and job termination [8], [31]. However, despite the fact that that ill health increases the risk of unemployment [32], [33], [34], neither of the studies of sick leave and unemployment attempted to adjust for the effect of the underlying disease.

The aim of this study was to investigate whether there is an association between long-term sick leave and disability pension and unemployment, when taking health status into account.

## Materials and Methods

This is a prospective cohort study based on data from the Stockholm Public Health Cohort (SPHC). The SPHC is based on random samples of the population of Stockholm County aged 18 to 84 years for participation in the Stockholm Public Health Surveys in 2002 and in 2007. In 2002 a total of 31,182 people responded to the self-administrated questionnaire (response rate 62.4%) and in 2007 these were reassessed in a further health survey in which 23,794 participated (retention rate 76%). The cohort also includes 1,028 individuals who answered the 2002 questionnaire but died before 2007 according to record linkage with the Cause of Death Register. Information from Swedish health and administrative registers was linked using the unique personal identity numbers. All information on exposure and confounders was taken from the 2002 questionnaire and the outcome information was taken from health and administrative registers. Written consent was obtained from all participants. The study has been approved by The Regional Ethical Review Board in Stockholm. The data collection and the present study conform to the principles of the Declaration of Helsinki.

At the time of this study all employees in Sweden were covered by the same sickness-benefit insurance, which after one qualifying day covered up to 80% of the income below a given limit, for full- or part-time sick leave. The first 14 days were financed by the employer, and thereafter by the Swedish Social Insurance Agency [35], [36], [37].

### Study sample

For the purpose of this study we restricted the cohort to encompass the 13,027 individuals (46% men) aged 18–59 in 2002, which were identified as being employed or self-employed and not unemployed, or on full or part-time disability pension. Characteristics of the study sample are presented in table 1.

**Table 1:** Characteristics of the study sample in 2002 (n = 13,027), n (%).

Background characteristics		Total	Long-term sick leave	No long-term sick leave	Missing information on sick leave
		(n = 13027)	(n = 1071)	(n = 11811)	(n = 145)
		n (%)	n (%)	n (%)	n (%)
Sex	Men	5996 (46.0)	352 (32.9)	5599 (47.4)	45 (31.0)
	Women	7031 (54.0)	719 (67.1)	6212 (52.6)	100 (69.0)
Age	18–29	1704 (13.1)	142 (13.3)	1545 (13.1)	17 (11.7)
	30–44	5328 (40.9)	375 (35.0)	4903 (41.5)	50 (34.5)
	45–59	5995 (45.0)	554 (51.7)	5363 (45.4)	78 (53.8)
Socio-economic position	Manual and skilled manual worker	3122 (24.0)	363 (33.9)	2700 (22.9)	59 (40.7)
	Low non-manual workers	1735 (13.32)	157 (14.7)	1556(13.2)	22 (15.2)
	Middle/high non-manual workers	6730 (51.7)	444 (41.5)	6235 (52.8)	51 (35.2)
	Self-employed	1231 (9.5)	83 (7.8)	1139 (9.6)	9 (6.2)
	Missing	209 (1.6)	24 (2.2)	181 (1.5)	4 (2.8)
Country of birth	Sweden	11214 (86.1)	854 (79.7)	10255 (86.8)	105 (72.4)
	Elsewhere	1770 (13.6)	211 (19.7)	1521 (12.9)	38 (26.2)
	Missing	43 (0.33)	6 (0.6)	35 (0.3)	2 (1.4)
Self-rated health	Very good/Good	10309 (79.1)	510 (47.6)	9742 (82.5)	57 (39.3)
	Fair/Bad/Very bad	2585 (19.8)	545 (50.9)	1955 (16.6)	85 (58.6)
	Missing	133 (1.0)	16 (1.5)	114 (1.0)	3 (2.1)
Mental health status (measured with GHQ 12)	Yes to <3 items	9976 (76.6)	624 (58.3)	9281 (78.6)	71 (49.0)
	Yes to≥3 items	2822 (21.7)	419 (39.1)	2331 (19.7)	72 (49.7)
	Missing	229 (1.8)	28 (2.6)	199 (1.7)	2 (1.4)
Limiting longstanding illness	No	11010 (84.5)	534 (49.9)	10402 (88.1)	74 (51.0)
	Yes, to a high degree/Yes, to some degree	1885 (14.4)	519 (48.5)	1296 (11.0)	70 (48.3)
	Missing	132 (1.0)	18 (1.7)	113 (1.0)	1 (0.7)
In-patient care during previous 12 months	Yes	592 (4.5)	211 (19.7)	365 (3.1)	211 (19.7)
	No	12435 (95.5)	860 (80.3)	11446 (96.9)	860 (80.3)
Self-reported somatic disease	Yes	464 (3.6)	78 (7.3)	374 (3.2)	12 (8.3)
	No	12427 (95.4)	972 (90.8)	11324 (95.9)	131 (90.3)
	Missing	136 (1.0)	21 (2.0)	113 (1.0)	2 (1.4)

### Exposure

Long term sick leave was defined as more than 30 sick-leave days in 12 months based on a survey question in 2002; *“If you have been on sick leave for more than 30 days during the previous 12 months, what was the main diagnosis?”* with response alternatives as, ‘Musculoskeletal illness’. ‘Mental illness/burnout’, ‘Allergic/respiratory illness’, ‘Skin illness’, ‘Cardiovascular illness’, and ‘Other’. In the analyses ‘Allergic/respiratory illness’, ‘Skin illness’, and ‘Other’ constituted one group ‘Others’. Those who had not been on sick leave at all or less than 31 days, during the previous 12 months were considered as unexposed.

### Outcomes

Two types of outcomes were used; disability pension and unemployment. Disability pension was assessed through register data from the Swedish National Social Insurance Agency and defined as a registered start date for disability during 2003–2007.

Unemployment was assessed based on the number of days registered as unemployed according to the Longitudinal Integration Database for Health Insurance and Labor Market Studies (LISA by Swedish acronym) during 2003–2006 (as 2007 was not available). Short-term unemployment was defined as having 1–180 registered days in one calendar year and long-term unemployment defined as having more than 180 days in one calendar year.

### Measures of health status

Information regarding four health indicators was retrieved from the 2002 survey and one from register information.

*Limiting longstanding illness* (LLSI) was based on two questions; “Do you have a longstanding illness, ailment after an accidental event, any handicap or other weakness?” with response alternatives “yes” or “no” and if yes, a supplementary question if this longstanding illness causes a reduced work capacity or limits one's other daily activities to a great extent, to some extent or not at all. LLSI was defined as having a longstanding illness that limited the work capacity to at least some extent.

*Somatic disease* was a combined measure including survey questions about whether the respondent ever had been diagnosed with any of the following diagnoses by a physician; diabetes, angina pectoris, myocardial infarction, heart failure, cerebral haemorrhage, and, additionally positive answers on questions regarding asthma and allergies during the past 12-month period.

*Self-rated health* (SRH) was measured by one survey question with five response alternatives from very bad to very good. Adverse self-rated health was defined as response alternatives; very bad, bad, and fair referred to as “less than good health”.

*Mental health* status was assessed using the General Health Questionnaire 12 (GHQ-12) [38] which was included in the survey. A score of three and above was considered as adverse mental wellbeing.

Data on days in *in-patient care* in during the 12 months period prior to answering the survey was based on information from the National Patient Registry, and dichotomized as any or no days.

### Socio-demographic variables

Information regarding potential confounders, socio-demographic and health indicators was retrieved from the 2002 survey responses and registers. The socio-demographic variables in 2002 were:

*Age* was categorized in three groups: 18–29, 30–44 and 45–59 years, and the younger group were used as reference category.

*Sex*, men and women, was measured by register data.

*Socio-economic status* (SES) was measured through a question on the respondent's occupation and classified in accordance with Statistics Sweden classification [39]. Each participant was allocated to one of the following four socio-economic groups: Higher and intermediate non-manual employees, lower non-manual employees, manual workers (skilled and unskilled), and self-employed (self-employed and farmers). Lower non-manual employees were used as the reference group.

*Country of birth* was based on a survey question, dichotomised as born in Sweden or elsewhere.

### Statistical Analyses

Descriptive analyses of background factors and potential confounders in the study group were computed from frequencies, and differences tested by Chi^2^ –tests (data not shown). For the outcome disability pension, the participants were followed until date of granted disability pension, death or end of follow-up (1^st^ of august 2008). For the outcome unemployment, the participants were followed until the first calendar year of having 1–180 registered unemployment days (or >180 unemployment days, respectively), death or end of follow-up (1^st^ of January 2007).

Hazard Ratios (HR) with corresponding 95% confidence intervals (CI) were estimated using Cox proportional hazards regression in order to model the effect of all cause and illness specific long-term sick leave on the risk of future disability pension and unemployment. In the analyses the effect was adjusted for health status and socio-economic situation in seven multivariate models; the first including socio-demographic characteristics, then adding each of the five measures of health status separately in the subsequent models, and finally the eighth full model including all socio-demographic and health status measures.

The illness-specific long-term sick leave was analysed for the following self-reported illnesses; ‘Musculoskeletal illness’, ‘Mental illness/burnout’, ‘Coronary-heart illness’, and ‘Other’ and adjusted for socio-demographic factors, SRH, and GHQ12.

All statistical analyses were performed using the SAS statistical software, version 9.1 (SAS Institute Inc, Cary, NC, USA).

## Results

The proportion of individuals exposed to long-term sick leave was higher among women, older, those born outside Sweden, and manual workers (table 1). Ill health, measured by self-reported health status (SRH, GHQ12, LLSI, somatic diseases) and by in-patient register data, was more common in the group with long-term sick leave than among those with no long-long term sick leave.

In the group on long-term sick leave in 2002, 18.6% had been granted disability pension during the follow-up period (table 2). Also, the percentage becoming unemployed was higher compared to those not on long-term sick leave. One third of persons on long-term sick leave due to musculoskeletal illness were granted disability pension and in this group the proportion of long-term unemployment (6.8%) was also the highest. The highest proportion of short-term unemployed was among people with mental illness (12.1%).

**Table 2:** Distribution of outcomes, stratified by illness-specific sick leave.

Exposure		Outcomes*		
Long-term sick leave	Total	Disability pension (n = 430)	Short-term unemployment (n = 896)	Long-term unemployment (n = 410)
No	11811 (100%)	231 (2.0%)	796 (6.7%)	351 (3.0%)
Yes (all illnesses together)	1071 (100%)	199 (18.6%)	100 (9.3%)	59 (5.5%)
Musculoskeletal illness	235 (100%)	71 (30.2%)	21 (8.96%)	16 (6.8%)
Mental illness	300 (100%)	64 (21.3%)	34 (11.3%)	20 (6.7%)
Coronary heart illness	28 (100%)	11 (39.3%)	1 (3.6%)	0 (0.00%)
Other illness	508 (100%)	53 (10.4%)	44 (8.7%)	23 (4.5%)

* The included outcomes are not mutually exclusive.

Individuals on long-term sick leave had a crude risk of disability pension which was more than 10 times higher than for those without long-term sick leave (table 3). Furthermore, long-term sick leave increased the crude risks of future short-term unemployment (HR 1.46; CI 1.19–1.80) and long-term unemployment (HR 1.89; CI 1.43–2.50). Adjustment for LLSI halved the HR of disability pension, and also decreased the HR of short-term unemployment (HR1.35; CI 1.08–1.70). SRH also decreased the HR of disability pension, although not as much as LLSI. The three self-reported measures of SRH, LLSI and GHQ12 had about the same effect on the HRs of short- and long-term unemployment, but GHQ12 decreased the HR of disability pension less than SRH and LLSI. Adjusting for socio-demographic factors decreased the effect estimates somewhat for disability pension and short-term unemployment but not for long-term unemployment.

**Table 3:** The HR (95% CI) of disability pension and unemployment among persons on long-term sick leave (LTSL) (n = 1071) in 2002, with adjustments for socio-demographic factors and health status step by step.

Regression models	Outcomes		
	Disability pension	Short-term unemployment	Long-term unemployment
	n = 390	n = 785	n = 364
	HR (95% CI)	HR (95% CI)	HR (95% CI)
No LTSL	1	1	1
Crude	10.94 (9.05–13.22)	1.46 (1.19–1.80)	1.89 (1.43–2.49)
Model 1: adjusted for socio-demographic factors^a^	9.36 (7.70–11.39)	1.34 (1.09–1.66)	1.88 (1.42–2.50)
Model 2: adjusted for SRH^b^	6.18 (5.05–7.56)	1.39 (1.12–1.73)	1.52 (1.14–2.04)
Model 3: adjusted for LLSI^c^	5.70 (4.62–7.03)	1.35 (1.08–1.70)	1.64 (1.22–2.21)
Model 4: adjusted for GHQ12^d^	10.07 (8.28–12.26)	1.34 (1.08–1.66)	1.68 (1.26–2.23)
Model 5: adjusted for somatic disease	10.55 (8.70–12.79)	1.40 (1.13–1.74)	1.88 (1.43–2.49)
Model 6: adjusted for in-patient care	10.54 (8.65–12.86)	1.43 (1.15–1.77)	1.98 (1.49–2.63)
Model 7: model 1+ adjusted for GHQ12, SRH, LLSI, somatic disease, in-patient care	4.01 (3.19–5.05)	1.18 (0.93–1.50)	1.45 (1.05–2.00)

a Socio-demographic factors: sex, age-groups, socio-economic status, country of birth.

b SRH = self-rated health.

c LLSI = Limiting long standing illness.

d Mental health status measured by GHQ12 [38].

The increased risk of disability pension varied depending on what illness the long-term sick leave was due to; from an adjusted HR of 10.13 (CI: 5.21–19.70) among those with coronary heart illness to an adjusted HR of 3.12 (CI:2.23–4.35) among those with other illness (table 4). There was also an increased risk of long-term unemployment among persons on sick leave for musculoskeletal and mental illness and this remained after adjustments. Further, an adjusted increased risk of short-term unemployment was seen among those on sick leave due to mental illness. Similarly to the analyses of all-cause exposure, adjustment for SRH reduced the HRs of disability pension more than adjustment for mental health status measured by GHQ12. Adjustment for mental health status also had less of an effect on the association between long-term sick leave due to musculoskeletal illness and unemployment.

**Table 4:** The HR (95% CI) of disability pension and unemployment among persons with illness-specific long-term sick leave (LTSL), with adjustments for socio-demographic factors, self-rated health (SRH), and mental health (measured with GHQ12).

Regression models	Disability pension (n = 390)	Short-term unemployment (n = 785)	Long-term unemployment (n = 364)
	HR (95% CI)	HR (95% CI)	HR (95% CI)
**Crude**			
No LTSL	1	1	1
- LTSL due to musculoskeletal illness	19.24 (14.74–25.10)	1.40 (0.91–2.16)	2.32 (1.40–3.84)
- LTSL due to mental illness	12.81 (9.71–16.90)	1.81 (1.28–2.54)	2.27 (1.45–3.57)
- LTSL due to coronary heart illness	30.55 (16.69–55.94)	0.51 (0.07–3.65)	-
- LTSL due to any other illness	5.80 (4.30–7.81)	1.34 (0.99–1.82)	1.57 (1.02–2.38)
**Model 1** a			
No LTSL	1	1	1
- LTSL due to musculoskeletal illness	13.59 (10.27–18.00)	1.28 (0.82–2.00)	2.00 (1.18–3.37)
- LTSL due to mental illness	12.84 (9.69–17.01)	1.81 (1.28–2.56)	2.48 (1.58–3.91)
- LTSL due to coronary heart illness	16.50 (8.73–31.20)	0.73 (0.10–5.20)	-
- LTSL due to any other illness	5.14 (3.79–6.97)	1.16 (0.85–1.58)	1.60 (1.05–2.45)
**Model 2** b			
No LTSL	1	1	1
- LTSL due to musculoskeletal illness	10.13 (7.68–13.37)	1.33 (0.86–2.06)	1.82 (1.09–3.04)
- LTSL due to mental illness	6.45 (4.82–8.63)	1.68 (1.17–2.39)	1.80 (1.13–2.85)
- LTSL due to coronary heart illness	19.11 (10.39–35.14)	0.50 (0.07–3.53)	-
- LTSL due to any other illness	3.67 (2.70–6.47)	1.31 (0.97–1.78)	1.30 (0.84–2.00)
**Model 3** c			
No LTSL	1	1	1
- LTSL due to musculoskeletal illness	17.48 (13.29–22.99)	1.39 (0.90–2.14)	2.26 (1.37–3.74)
- LTSL due to mental illness	11.13 (8.32–14.89)	1.55 (1.09–2.19)	1.89 (1.19–2.99)
- LTSL due to coronary heart illness	29.92 (16.34–54.78)	0.51 (0.07–3.59)	-
- LTSL due to any other illness	5.65 (4.20–7.63)	1.24 (0.91–1.69)	1.36 (0.88–2.12)
**Model 4** d			
No LTSL	1	1	1
- LTSL due to musculoskeletal illness	7.50 (5.59–10.07)	1.26 (0.80–1.98)	1.70 (1.00–2.89)
- LTSL due to mental illness	6.89 (5.07–9.34)	1.57 (1.09–2.26)	1.80 (1.13–2.88)
- LTSL due to coronary heart illness	10.13 (5.21–19.70)	0.66 (0.09–4.73)	-
- LTSL due to any other illness	3.12 (2.23–4.35)	1.06 (0.77–1.47)	1.27 (0.79–2.03)

a adjusted for socio-demographic factors.

b Model 2 adjusted for self-rated health (SRH).

c Model 3: adjusted for mental health status measured with GHQ12 [38].

d Model 3: adjusted for socio-demographic factors, SRH, GHQ12 and in-patient care 12 months before baseline.

## Discussion

In our study sample, exposure to long-term sick leave in 2002 was more common among women, older, manual workers, and people born outside Sweden, than among men, younger, non-manual workers, and people born in Sweden. Persons exposed to long-term sick leave had an risk of all three outcomes, disability pension, short-term unemployment and long-term unemployment. An increased risk remained after adjustments for health status except for the outcome short-term unemployment which did not remain statistically significant after adjustment. SRH changed the HR of disability pension and long-term unemployment more than other health indicators. Mental health status measured by GHQ12 had an impact on the risk of unemployment but not disability pension.

Stratifying the exposure of long-term sick leave by type of illness indicated an increased risk of disability pension irrespective of which illness the sick leave was due to. For long-term unemployment, however, there was only a statistically significant increased risk among individuals with sick leave due to musculoskeletal and mental illness. Few individuals had sick leave due to coronary hearth illness, which resulted in HRs with wide confidence intervals and in the case of long-term unemployment no effect estimate at all.

Previous studies of risk factors of disability pension have focused either on identifying work- or demography- or health-related predictors [6], [12], [16], [17], [18], [20], [23], [24], [25], [40], [41]. The work-related predictors can, just as measures of health behavior, be expected to have their main effect through the emergence of ill health and subsequent long-term sick leave, although this is rarely explicitly investigated.

Pietiläinen et al [23] has shown that different health-related predictors capture different aspects of the relationship between ill health and disability pension. Long-term sick leave is one established health-related predictor of disability pension [5], [6], [12], [13], [14], [20]. The question is, is it just an indicator of ill health or does long-term sick leave have consequences of its' own? The presented results indicate that there is an association between long-term sick leave and disability pension even after adjustment for SRH, LLSI, GHQ12 and inpatient care.

Previous studies of sick leave and unemployment are scarce. The few which exist indicate an association between the two [8], [30]. However these studies do not take ill health into account, which has been shown to be associated with unemployment [32], [33], [34]. Our results indicate that long-term sick leave has an effect on the risk of future long-term unemployment beyond the effect of health status.

Taken together, the results indicate that long-term sick leave may start a process of labor-market marginalization, which have been suggested previously by Bryngelsson [29].

### Strengths and Limitations

The main strengths of this study is the longitudinal design, the large population-based study group and the opportunity to employ both self-reported and register data.

The associations between long-term sick leave and disability pension and unemployment are extensively adjusted for both self-reported and register-based measures of ill health. This captured different aspects of health, measured by the generic measures SRH and LLSI, and for common mental health GHQ-12. The GHQ-12 questionnaire is a well-established and validated instrument, measuring mental well-being [38]. Furthermore, the questions covering limiting long-standing illness and specified somatic diseases capture the most common somatic diagnosis among sickness absent, e.g. musculoskeletal diseases. The access to registered in-patient care made it possible to control for those with diseases like tumors, psychiatric diseases, and coronary heart diseases that required in-patient care for shorter or longer periods during the 12 months before answering the survey. This 12-month period is consistent with the period for which long-term sick leave was reported in the questionnaire. These adjustments, together with the adjustments for socio-demographic factors should handle the possibly confounding effects of work characteristics and health behavior reported in previous studies [12], [16], [24], [25], [40], [41], [42]. However, it should be noted that it is not possible to fully discriminate between the consequences of sick leave and the ill-health, as we cannot link the health status with each specific sick-leave spell. Furthermore, the measurement of the specific illnesses is somewhat crude and it cannot be excluded that the individuals exposed to long-term sick leave may also have been absent due to other illness than reported.

The social security system in Sweden, which implies that the first two weeks of sick leave is compensated by the employer, also means that no register data is available on the total number of sick-leave days and spells. However, the used measure of self-reported sick leave of more than 30 days in total one year was compared with data from the Swedish National Social Insurance Agency of sick-leave spells longer than 30 days for the same period. The results indicated that 1.1% of those reporting not having had long-term sick leave were classified as unexposed although they according to the register had a sick-leave spell of at least 30 days (data not shown). Such possible misclassification may dilute the effect estimates. Specificity cannot be explored with a similar comparison since register-based data do not include shorter spells.

The Swedish social security system also implies that most disability pensioners have gone through a period of long-term sick leave before being granted disability pension. In this study we define exposure in a limited window in time that is, twelve months prior answering the initial questionnaire in 2002, and it is possible, and likely, that some individuals who have been classified as unexposed may later have experienced long-term sick leave spells. Such possible misclassification of exposure may dilute the estimated effects.

### Conclusions

Long-term sick leave seems to increase the risk for disability pension and unemployment independently of health status. It seems that the specific illness for which sick leave is taken is of importance for the associations. This study supports the hypotheses that long-term sick leave may start a process of marginalization from the labor market.
